# How do Paraspinal Muscles Contract during the Schroth Exercise Treatment in Patients with Adolescent Idiopathic Scoliosis (AIS)?

**DOI:** 10.3390/bioengineering9060234

**Published:** 2022-05-27

**Authors:** Chen He, Jian-Tao Yang, Qian Zheng, Zhao Mei, Christina Zong-Hao Ma

**Affiliations:** 1 Institute of Rehabilitation Engineering and Technology, University of Shanghai for Science and Technology, Shanghai 200093, China; hechen@usst.edu.cn; 2 Department of Rehabilitation Medicine, Tongji Hospital, Tongji Medical College, Huazhong University of Science and Technology, Wuhan 430030, China; qianzhengtongji@163.com; 3 Department of Technology, Shanghai Huazhu Medical Institution, Shanghai 201204, China; meizhao2003@163.com; 4 Department of Biomedical Engineering, The Hong Kong Polytechnic University, Hong Kong SAR 999077, China; czh.ma@polyu.edu.hk

**Keywords:** adolescent idiopathic scoliosis (AIS), surface electromyography (sEMG), paraspinal muscle, Schroth exercise, paraspinal muscle symmetry index (PMSI)

## Abstract

The Schroth exercise can train the paraspinal muscles of patients with adolescent idiopathic scoliosis (AIS), however, muscle performance during the training remains unknown. This study applied surface electromyography (sEMG) to investigate the paraspinal muscle activities before, during and after Schroth exercise in nine AIS patients. This study found that after the Schroth exercise, the paraspinal muscle symmetry index (PMSI) was significantly reduced (PMSI = 1.3), while symmetry exercise significantly lowered the PMSI (PMSI = 0.93 and 0.75), and asymmetric exercise significantly increased the PMSI (PMSI = 2.56 and 1.52) compared to relax standing (PMSI = 1.36) in participants (*p* < 0.05). Among the four exercises, the PMSI of on all fours (exercise 1) and kneeling on one side (exercise 3) was the most and the least close to 1, respectively. The highest root mean square (RMS) of sEMG at the concave and convex side was observed in squatting on the bar (exercise 2) and sitting with side bending (exercise 4), respectively. This study observed that the asymmetric and symmetric exercise induced more sEMG activity on the convex and concave side, respectively, and weight bearing exercise activated more paraspinal muscle contractions on both sides of the scoliotic curve in the included AIS patients. A larger patient sample size needs to be investigated in the future to validate the current observations.

## 1. Introduction

Scoliosis is a three-dimensional deformity of the lateral curvature and rotated vertebrae, among which adolescent idiopathic scoliosis (AIS) is the most commonly diagnosed. The prevalence of AIS is reported as high as 1.02–2.4% among primary and secondary school students [1,2]. The deformed spine in patients with AIS leads to asymmetric paraspinal muscles that show higher electromyographic (EMG) activity on the convex side than that of the concave side of the scoliotic curve [3,4,5]. This asymmetry could be due to a lower proportion of oxidative slow-twitch (type 1) fibers on the concave side, which induced a decrease in tonic activity and the ability to sustain contractions, resulting in sustained postural deficits [5,6]. The imbalance and asymmetry in the paraspinal muscles have been suggested to be related to the development and progression of spinal deformity [4] and decreased quality of life in AIS patients [7], which warrants further efforts to identify and validate the appropriate treatment.

Treatment of the musculature is one of the main objectives in AIS, as the effect of the correction in posture needs to be maintained by the musculature. Different treatment exercises have been proposed, and the muscle responses to these exercises have been investigated in a number of previous studies. Schmid et al. (2010) assessed the surface electromyography (sEMG) activity of paraspinal muscles during four back strengthening exercises on patients with AIS, and found that the asymmetric exercises of the front press at the lumbar level and the roman chair and bent-over barbell row at the thoracic level were superior in increasing sEMG amplitudes in the concave side [8]. Chwala et al. (2014) compared sEMG activity symmetry during two symmetric and four asymmetric exercises in girls with AIS, and found that most cases generated an increase in the predominance of sEMG activity at the convex side during symmetric and asymmetric exercises than in the resting position [9]. Strasse et al. (2018) validated the application of sEMG in monitoring the neuromuscular activity after an exercise treatment lasting for 12 weeks. They found improved balance in the recruitment of motor units for the production of muscle strength after exercise, especially at the right side of the spine [10]. Tsai et al. (2010) investigated the difference in bilateral paraspinal muscle activities during resistance isokinetic exercises in people with and without scoliosis [11]. They found that the paraspinal muscle tended to shift sEMG activities from the convex to the concave side, and the lumbar paraspinal muscle supplied the major action in healthy subjects, while thoracic paraspinal muscle compensated to supply actions in patients with a larger scoliosis curve. As a result, they recommended more midback protection during exercises on patients with AIS.

The Schroth exercise is a common approach for paraspinal muscle training for patients with AIS in clinical practice. It was developed by Katharina Schroth in 1920 [12]. The scoliosis-specific exercise is specifically designed to train patients to bring their asymmetric posture into alignment and restore a correct upright position. The repetitive training of the skeletomuscular system could reinforce the effect, so that patients could consciously maintain the corrected posture in daily living activities [13]. The Schroth method also provides sensorimotor and breathing exercises aimed at the recalibration of static/dynamic postural control, spinal stability, and breathing patterns [14]. It has been reported that the Schroth exercise slows curve progression [15], reduces curve severity [16] and reduces scoliosis related pain (>50% intensity and frequency) [17]. Furthermore, the Schroth exercise was also reported to improve the performance capacity of the paraspinal muscles, such as strengthening the musculature, better exploiting muscle activity [12], improving erector spinae activation strategies [6], and correcting the postural defects [18]. These benefits may be presented by a more symmetric sEMG activity on the concave and convex side of the paraspinal muscles. 

However, to the best of the authors’ knowledge, few previous studies have investigated the sEMG activity while performing the Schroth exercise in patients with AIS. Therefore, this study aimed to address this issue and applied sEMG innovatively to investigate the paraspinal muscles activity before, during and after the Schroth exercise in AIS patients. The muscle performance in the Schroth exercise, as revealed via sEMG signal, will provide evidence and contribute to the individualized and case-specific training of the Schroth exercise for patients with AIS in future clinical practice.

## 2. Materials and Methods

### 2.1. Participants

Patients with AIS were recruited through the outpatient clinic specializing in the treatment of scoliosis. The inclusion criteria were: (1) diagnosed as AIS; (2) 10–18 years old with Risser sign ranged from 0 to 5; (3) Cobb angle between 20°~50°; and (4) experienced in the Schroth exercise (i.e., received the Schroth exercise training for at least three times previously) to control and ensure a good exercise performance among the participants. A sample size of 21 subjects was calculated (assuming that the effect size (d) = 0.5; statistical power (1 − β) = 0.8; level of significance (α) = 0.05 used for 2-tailed T-test). Patients with severe back pain, spinal surgery history and other neurological symptoms were excluded from this study. All participants gave their written informed consent to participate in the study. 

### 2.2. Assessment Procedure

A Noraxon EMG assessment system with wireless electromyography sensors (Noraxon Inc., Scottsdale, AZ, USA) was used for data collection. The sampling rate of the EMG Sensor System was 1500 Hz with ±24,000 μV EMG input range. The baseline noise was less than 1 μV. The selectable low-pass cut-off and high-pass cut-off were at 500/1000/1500 Hz and 5/10/20 Hz, respectively. The CMRR of EMG preamplifier was more than 100 dB.

#### 2.2.1. Before Exercise

Each participant was instructed to stand in a relaxed position. Surface electrodes were placed on the skin surface of superficial erector spinae muscles, 3-cm from the midline and parallel to the spinous processes of the apical vertebra. The level of the apical vertebra was determined on the radiographs by an experienced clinician. Then, the sEMG signals were recorded for 20 s during the relaxed standing position. The test was repeated three times, with at least 3 min of rest in between to minimize the effect of fatigue in participants.

#### 2.2.2. During Exercise

**Figure 1:**
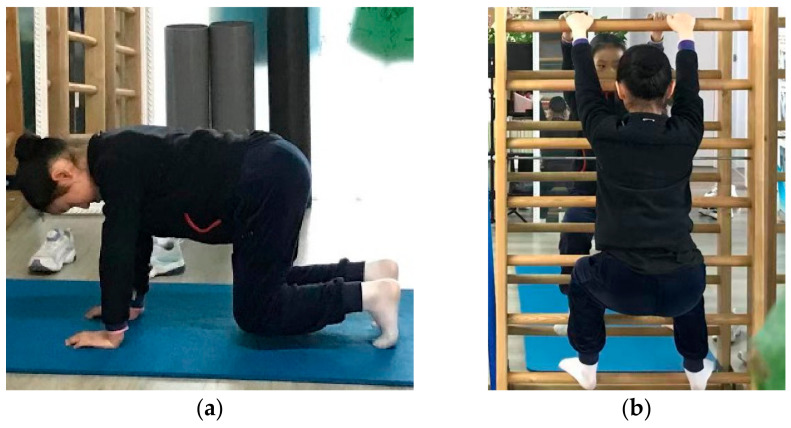

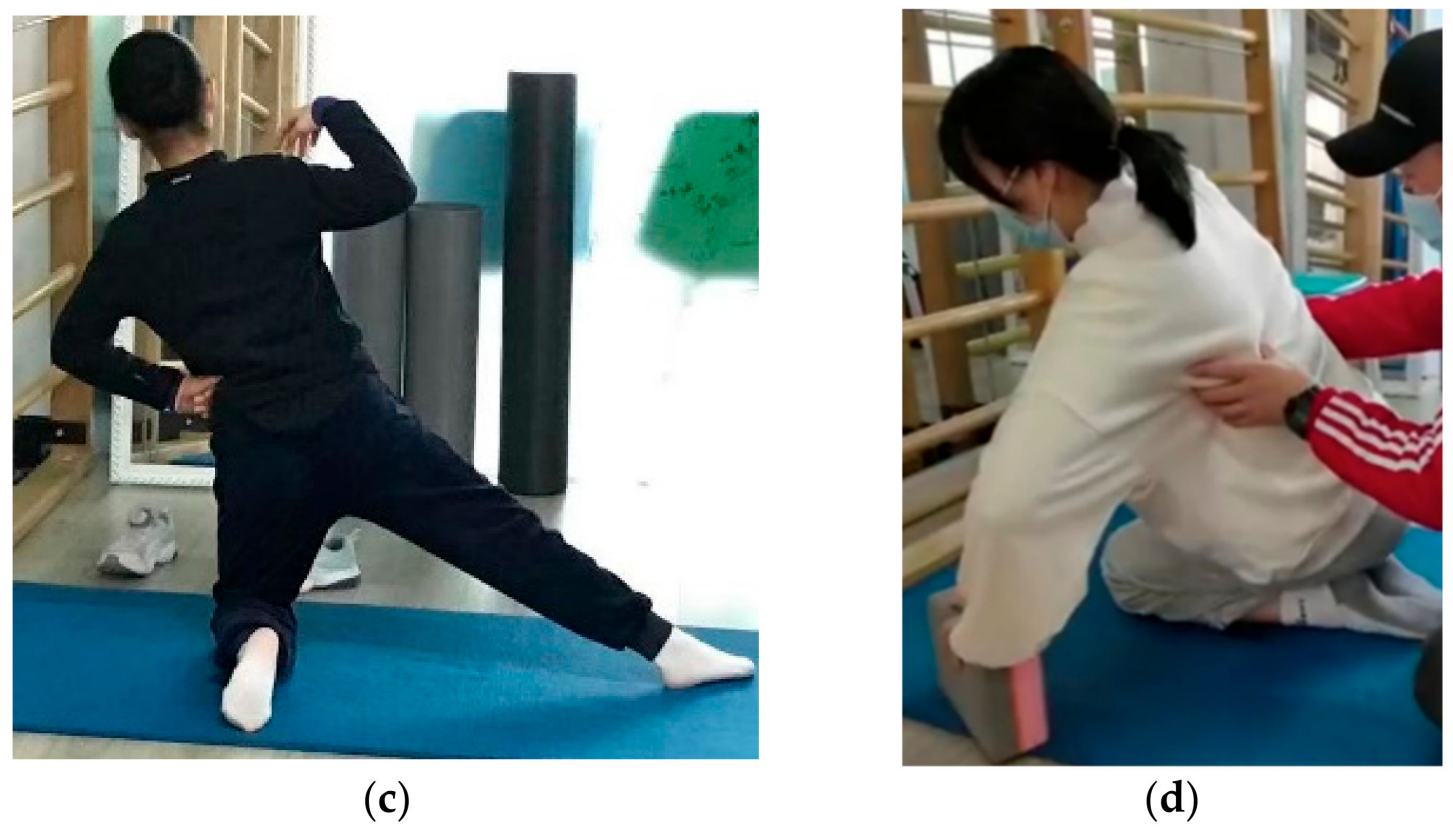
Four different Schroth exercise positions: (**a**) E1—on the fours, (**b**) E2—squatting on the bar, (**c**) E3—kneeling on one side, (**d**) E4—sitting with side bending.

A physiotherapist specializing in the Schroth exercise instructed participants in performing the exercises. The sEMG data was collected in four different exercise positions, which were performed in a randomized order (Figure 1). Exercise 1 (E1) and exercise 2 (E2) were symmetric exercises, and exercise 3 (E3) and exercise 4 (E4) were asymmetric exercises. It is routine practice that each exercise is repeated three to five times in the clinic as specified in Lehnert-Schroth (2007) [12]. Thus, each test was repeated three times, aiming to minimize the influence of a daily training schedule and reflect the muscle performance in a real clinical situation. Furthermore, rest for at least 3 min in between was allowed to minimize the effects of fatigue in participants. Details of each exercise position are provided below.

E1 (on the fours): The participant kneeled down with the knees apart at shoulder width and kept the thighs in a vertical position. The arms were extended vertically under the shoulders to support the body, with the fingers pointing straight ahead [12]. The participant kept a steady breath and sustained this position for 20 s, during which the sEMG signal was recorded. Each participant repeated this procedure three times to acquire an average sEMG value.

E2 (squatting on the bar): The participant put the feet on the second bar, and the hands apart on bar at shoulder level in a squatting position. The participant would then guide the hip below the rib hump to move laterally, backwards, and downward [12]. Then the participant sustained the downward position for 20 s, during which the sEMG signal was recorded. Each participant repeated this procedure three times to acquire an average sEMG value.

E3 (kneeling on one side): The participant kneeled down with the trunk leaning over to the convex side, then stretched out the leg on the concave side, rotated outwards and placed it laterally to form the leg and the upper body as a line. The participant kept the pelvis upright and hands on the hips. The participant kept a steady breath and sustained this position for 20 s, during which the sEMG signal was recorded. Each participant repeated this procedure three times to acquire an average sEMG value. 

E4 (sitting with side bending): The participant sat with the buttock on the heel and kept the pelvis upright, then leaned the trunk over to the convex side and put the hand on the convex side on a block to support the oblique body. Then, the participant sustained this position for 20 s, during which the sEMG signal was recorded. Each participant repeated this procedure three times to acquire an average sEMG value.

#### 2.2.3. After Exercise

The participant was instructed to stand in the relaxed standing position. The same procedure of sEMG activity measurement as the pre-exercise was taken again, to record the sEMG signals after exercise.

### 2.3. Data and Statistical Analysis

The obtained sEMG signals were amplified and sampled at 1500 Hz using myoMUSCLE™ software (Noraxon Inc., Scottsdale, AZ, USA). The raw data was band-passed filtered (Butterworth with a cut-off frequency of 20–500 Hz). The sEMG signal of each exercise was divided into three sequences. Each sequence was normalized for time. The root mean square (RMS) quantifying the sEMG amplitude of the averaged sEMG signal was calculated. The paraspinal muscle symmetry index (PMSI) was calculated as RMS_convex_/RMS_concave_. The PMSI of being close to 1 (e.g., PMSI = 1) referred to the high symmetry of the paraspinal muscle. The PMSI < 1 referred to a greater RMS_concave_ than RMS_convex_, and PMSI > 1 referred to a greater RMS_convex_ than RMS_concave_ of the scoliotic curve. 

The statistical package SPSS, version 22 (SPSS Inc, Chicago, IL, USA), was used for all statistical analyses. One-way repeated ANOVA was used to compare the PMSI before, during and after exercise, and examine for the existence of significant difference. A post hoc adjusted for multiple comparisons with the Bonferroni method was used if significant differences among overall PMSIs were found. Two-way repeated ANOVA and the Bonferroni correction for multiple comparisons was adopted to analyze the RMS of sEMG on the concave and convex side before, during and after exercise, and examine for the existence of significant difference. The level of significance was set at 0.05. 

## 3. Results

### 3.1. Participants

A total of nine patients with AIS participated in this study. Their demographic data are shown in Table 1.

### 3.2. The PMSI and RMS of sEMG Activity of Paraspinal Muscles

The PMSI and RMS values of sEMG activity of paraspinal muscles are shown in Table 2. The PMSI of pre-exercise and post-exercise in the relaxed standing position was over 1, which meant the sEMG activity of the paraspinal muscle on the concave side was lower than that of the convex side. The PMSI significantly reduced from 1.36 to 1.30 after exercise (*p* < 0.05), indicating the sEMG activity symmetry of the paraspinal muscle between the convex and concave side was improved.

#### 3.2.1. The PMSI before, during and after the Schroth Exercise

**Figure 2:**
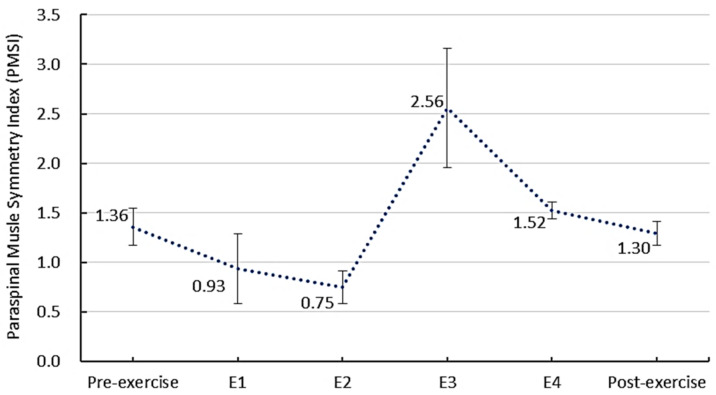
The PMSI before, during and after the Schroth exercise (*n* = 9).

The PMSI values before, during and after the Schroth exercise are shown in Figure 2. The PMSI during E1 reduced significantly to 0.93 from 1.36 (*p* < 0.05) in the relaxed standing position. It suggested that the sEMG activity of paraspinal muscle at the concave side increased and reached a similar level to that of the convex side, which improved the symmetry of the paraspinal muscles. The PMSI during E1 was closest to 1 among the four exercises, with no significant difference between the RMS_concave_ and the RMS_convex_; thus, it may be regarded as the exercise with the highest symmetry of the sEMG activity among the four exercises. The PMSI during E2 was reduced to 0.75 from 1.36 (*p* < 0.05) in the relaxed standing position. This suggests that the sEMG activity of paraspinal muscle on the concave side increased and reached to the level closer to the convex side, which improved the symmetry of the paraspinal muscle. The RMS_concave_ and the RMS_convex_ during E2 did not show significant difference. 

The PMSI during E3 increased significantly to 2.56 from 1.36 (*p* < 0.05) in the relaxed standing position. This suggested that the sEMG activity of paraspinal muscle at both sides increased but the convex side increased more, which reduced the symmetry of the paraspinal muscle. The PMSI during E3 was the least close to 1 among the four exercises, with the sEMG activity of convex side being significantly higher than that of the concave side (*p* < 0.05). Therefore, it may be regarded as the exercise with the lowest symmetry among the four exercises. The PMSI during E4 increased significantly to 1.52 from 1.36 (*p* < 0.05) in the relaxed standing position. This suggests that the sEMG activity of the paraspinal muscle on both sides increased but the convex side increased more, which reduced the symmetry of the paraspinal muscle. 

#### 3.2.2. The RMS of sEMG Activity before, during and after the Schroth Exercise

**Figure 3:**
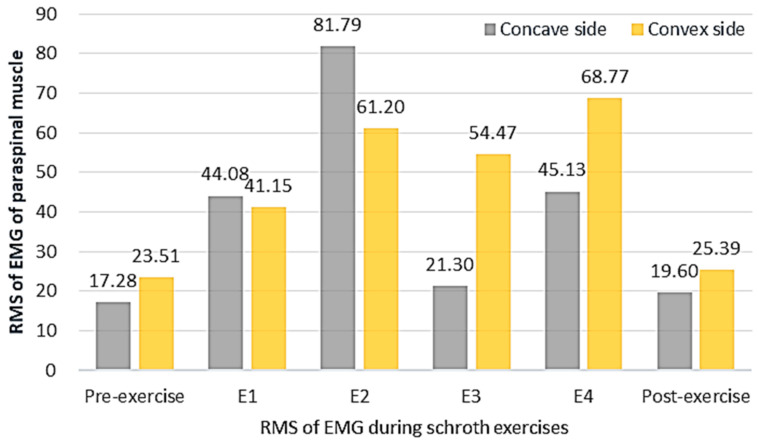
The RMS of sEMG before, during, and after the Schroth exercise (*n* = 9).

The RMS of sEMG before, during, and after the Schroth exercise is shown in Figure 3. The sEMG activity of paraspinal muscle was higher in all Schroth exercises than that in the relaxed standing position before exercise. The RMS_concave_ significantly increased after exercise (23.51 μV vs. 25.39 μV, *p* < 0.05), while the RMS_convex_ did not significantly change after exercise (17.28 μV vs. 19.60 μV, *p* > 0.05), which indicates that the exercise induced more sEMG activity of paraspinal muscle change on the concave side of the scoliotic curve.

The highest RMS_concave_ (81.79 μV) was observed in E2, which was a symmetric exercise against gravity and induced muscle contraction on both sides. The highest RMS_convex_ (68.77 μV) was observed in E4, which was an asymmetric exercise, with side bending to the convex side and stretching of the concave side. Upon comparing the magnitude of sEMG activity of paraspinal muscles, this study observed that E4 > E3 (*p* < 0.05) and E2 > E1 (*p* < 0.05) in both the convex and concave side of the scoliotic curve.

## 4. Discussion

This study innovatively applied the sEMG to investigate paraspinal muscle activities before, during and after the Schroth exercise in patients with AIS. The findings of this study will provide evidence and support the individualized and case-specific prescription of the Schroth exercise in future clinical practice, which could improve the effectiveness of treatment and improve the quality of life of patients with AIS.

The sEMG activity of the paraspinal muscle on the concave side was found to be lower than that of the convex side during the relaxed standing position in AIS patients. This could be explained by the prolonged stretching of the paraspinal muscles due to deformed vertebrae in AIS patients, which resulted in the asymmetry of muscle fiber types, lengths and locations at bilateral sides [5,19]. The indicated functional imbalance of the paraspinal muscles in AIS suggests that clinicians should prescribe specific exercise to improve muscle balance on both sides according to individual conditions. 

This study found that the PMSI of AIS reduced by 4.6% after the Schroth exercise. A previous study also reported the reduced PMSI by 12.0% in the thoracic region and by 7.9% in the lumbar region after the Schroth exercise [6]. Since only patients with single lumbar scoliosis were recruited in the current study, the influence of curve location on the symmetry of paraspinal muscles could be investigated in future studies. 

During symmetric exercises, the sEMG activity of the paraspinal muscle was symmetric, while during the relaxed standing position was asymmetric. Chwala et al. [9] also reported higher PMSI during symmetric exercise in comparison with the resting recordings. The possible reason could be that symmetric exercise tried to isolate the muscle contraction between the concave and convex side, and focused more on the atrophied concave side to improve the symmetry of paraspinal muscles [18]. For E1 (on the fours), the sEMG activity on the concave side increased more than the convex side and reached a symmetric sEMG activity on both sides. E1 may be regarded as a symmetric exercise with reduced longitudinal gravity on the spine, which would simultaneously correct the sagittal lordosis and coronal scoliosis of spinal deformity [20]. The symmetric sEMG activity of paraspinal muscles during E1 may be related to both the self-correction of the patients and the spontaneous correction by the postural change. This can also explain the highest RMS_concave_ in E2 (squatting on the bar), which is a symmetric exercise that was against gravity and induced higher muscle contraction on both sides.

During asymmetric exercises, the sEMG activity of paraspinal muscle on the concave side was lower than that of the convex side. The paraspinal muscle fiber was reported to be weaker on the concave side and stretched on the convex side in the scoliotic spine [21]. The convex side was usually used as the dominant side for daily activities. During asymmetric exercise, side bending created an imbalance load on the spine, requiring greater paraspinal muscle contraction to maintain stability. As a result, an increase in the predominance of the sEMG activity on the convex side was instigated. It could be a sign of an adaptive response to the greater use of the muscles on the convex side in patients with AIS. The highest RMS_convex_ was observed in E4 (sitting with side bending), which agreed with Chwala et al.’ s study [9] who observed the highest sEMG activity of the convex side of paraspinal muscles in an asymmetric exercise, which involved actively stretching the concave side. They also reported that asymmetric exercises demonstrated larger differences in sEMG activity of the paraspinal muscles in comparison with symmetric exercises.

When considering individual patients, two out of nine patients demonstrated lower sEMG activity at the concave side during symmetric exercise, which was opposite to the other subjects. This might be because each patient had different motor habits and variable attempts when performing exercises. The same exercise could result in diverse performance quality and repeatability of the corrective patterns in practice [9]. Therefore, individualized exercise should be recommended based on the specific muscle response and performance quality of patients. This study validated the feasibility of applying sEMG to evaluate the muscle performance during the Schroth exercise, which will provide evidence and contribute to the case-specific training for patients with AIS in clinical practice. It may also be helpful to adopt some ultrasound imaging technologies [22] to study the internal paraspinal muscle contraction pattern during the exercise in AIS patients in the future.

This study has several limitations. This study only involved nine patients with lumbar scoliosis. A larger sample size with diverse types of scoliosis curve needs to be investigated. Unfortunately, due to the COVID-19 pandemic in China, it is extremely difficult to recruit more AIS patients for this study at this time and in the near future. The current study may serve as pilot investigation providing the theoretical foundation and research direction for future studies to further validate the current observations and deepen the knowledge in this field with larger samples. This study has focused on the immediate effect of the exercise on the paraspinal muscles, but lengthier studies will be necessary to confirm the long-term effects of the Schroth exercise on the performance of the paraspinal muscles. It would also be interesting to investigate whether any difference existed in paraspinal muscle activity during the Schroth exercise between adolescents with and without scoliosis. However, due to the limited number of available children/adolescent participants, it has been difficult to recruit the healthy adolescents without scoliosis to perform the Schroth exercise as a control group, especially under the current pandemic situation. Future studies could recruit some healthy children/adolescents without scoliosis to study the difference in paraspinal muscle activity during the Schroth Exercise.

## 5. Conclusions

This study observed that the sEMG activity of paraspinal muscle was higher during Schroth exercise than in that of a relaxed standing position in nine patients with AIS. The asymmetric exercise induced more sEMG activity at the convex side, while symmetric exercise induced more sEMG activity at the concave side. Weight bearing exercise tended to activate more muscle contractions on both sides of the scoliotic curve in the included AIS patients. Patients in a larger sample size will need to be investigated in the future to validate the current observations.

## Figures and Tables

**Table 1:** Demographic data (*n* = 9).

Demographic Data	Description
Age	15.2 ± 3.3 years
Gender	9 females
Body Mass Index (BMI)	18.56 ± 1.66
Cobb Angle	31.56° ± 8.29°
Curve Type	C curve
Apex	T7~L2
Risser Sign	0~5

**Table 2:** Paraspinal muscle symmetry index (PMSI) before, during and after Schroth Exercise (*n* = 9).

	RMS_concave_ (μV)	RMS_convex_ (μV)	PMSI	*p* Values
Pre-exercise	17.28 ± 6.18	23.51 ± 6.55	1.36 ± 0.19 *	<0.01
E 1	44.08 ± 14.58	41.15 ± 20.49	0.93 ± 0.35	0.08
E 2	81.79 ± 24.01	61.20 ± 44.59	0.75 ± 0.16	0.06
E 3	21.30 ± 15.72	54.47 ± 5.37	2.56 ± 0.60 *	<0.01
E 4	45.13 ± 21.19	68.77 ± 16.51	1.52 ± 0.09 *	<0.01
Post-exercise	19.60 ± 6.17	25.39 ± 5.34	1.30 ± 0.12 *	0.03

* The RMS_concave_ and the RMS_convex_ were significantly different (*p* < 0.05).

## Data Availability

Data are presented in the article. Initial instrumental output data are available upon request from corresponding author.
